# Comparative genomic analysis uncovers candidate genes related with milk production and adaptive traits in goat breeds

**DOI:** 10.1038/s41598-023-35973-0

**Published:** 2023-05-30

**Authors:** Zeinab Amiri Ghanatsaman, Ahmad Ayatolahi Mehrgardi, Hojjat Asadollahpour Nanaei, Ali Esmailizadeh

**Affiliations:** 1 Department of Animal Science, Faculty of Agriculture, Shahid Bahonar University of Kerman, Kerman, 76169-133 PB Iran; 2 Animal Science Research Department, Fars Agricultural and Natural Resources Research and Education Center, Agricultural Research, Education and Extension Organization (AREEO), Shiraz, Iran; 3 Key Laboratory of Animal Genetics, Breeding and Reproduction of Shaanxi Province, College of Animal Science and Technology, Northwest A&F University, Yangling, 712100 China

**Keywords:** Animal breeding, Genetics, Comparative genomics

## Abstract

During the process of animal domestication, both natural and artificial selection cause variation in allele frequencies among populations. Identifying genomic areas of selection in domestic animals may aid in the detection of genomic areas linked to ecological and economic traits. We studied genomic variation in 140 worldwide goat individuals, including 75 Asian, 30 African and 35 European goats. We further carried out comparative population genomics to detect genomic regions under selection for adaptability to harsh conditions in local Asian ecotypes and also milk production traits in European commercial breeds. In addition, we estimated the genetic distances among 140 goat individuals. The results showed that among all studied goat groups, local breeds from West and South Asia emerged as an independent group. Our search for selection signatures in local goats from West and South Asia revealed candidate genes related to adaptation to hot climate (*HSPB6*, *HSF4*, *VPS13A* and *NBEA* genes) and immune response (*IL7*, *IL5*, *IL23A* and *LRFN5*) traits. Furthermore, selection signatures in European commercial goats involved several milk production related genes, such as *VPS13C*, *NCAM2*, *TMPRSS15*, *CSN3* and *ABCG2*. The identified candidate genes could be the fundamental genetic resource for enhancement of goat production and environmental-adaptive traits, and as such they should be used in goat breeding programs to select more efficient breeds.

## Introduction

It is believed that domestic goats have been originated from the wild bezoar in the Fertile Crescent and adjacent area^1^. Their domestication process started about ten thousand years ago in the Neolithic period, just after people's style of living shifted from hunting to farming^2^. Since then, domestic goat provided hair, fur, meat and milk for human consumption^3^. Following human migration and trade activities over the past thousand years, domestic goats have been adapted to the environmental conditions under which they have been reared^4,5^. Today, they comprise over 300 breeds and more than 1006 million individuals, covering indigenous and commercial breeds (http://faostat3.fao.org/browse/Q/QA/E). Throughout the globe, domesticated goats have been adapted to different climate conditions^6^. For example, in arid regions of Morocco, native goat breeds have acquired traits related to heat tolerance^7^. In high altitudes of Tibetan Plateau native goats have adapted to the local conditions (such as low-oxygen)^8^. Moreover, Ugandan native goats have raised their immune merit as a means to abide contamination via parasites in Africa’s hot condition^9^. These acclimated livestock have prepared a main base for different breeding strategies intended improving selective targets^10^. For instance, Chinese native goats in the Shandong area have crossed with Saanen dairy goats^11^ to create Laoshan dairy goats. Due to this attempt, the Laoshan dairy goats have improved to produce high dairy profits and also acquired adaptation to wet weather condition in local area^12^. In addition, Indonesian native goats in the tropical weather have crossed with Indian native goats^11^ to create Peranakan Etawah goats. Previous studies have shown that this breed has higher production capacity for producing milk and meat and also adapted to tropical weather conditions^13^. These studies collectively show that local breeds can survive in harsh conditions and have developed immunity to diseases prevalent in the local conditions.

Over the last few decades, universal climate data perspicuously shows a warming tendency in nearly all areas of the globe, consequent to an extensive dimension of weather changes^14^. Recently, a considerable diversity of environmental adaptation traits has been detected in different species such as sheep^15^, goats^15,16^, chickens^17,18^ and cattle^19–21^. The current global goat population is 1.002 billion, chiefly existing in Asia (57.7%) and Africa (35.7%), as reported by the Food and Agriculture Organization (FAO), comprising 93.4% of the total number in the world, which has doubled in the last thirty years^22^. The outstanding countries in goat production included five Asian (China, India, Pakistan, Bangladesh and Iran) and five African (Nigeria, Sudan-former, Sudan, Kenya and Ethiopia) countries^22^.

Genetic variation in livestock has been acknowledged as a main feature. The preservation of genetic variation is essential in livestock for increasing yield and for answering future issues, which consist of changing environments and food safety^23^. Long-period natural selection in native goats has led to alterations in the allele frequency and so desirable adaptability as an effect of evolvement, however, commercial goat breeds have been created via a series of extensive artificial selection for increasing production traits. As well, selection signatures identified in domestic animals increase awareness about population demography and will disclose the basis of phenotypic diversity among livestock breeds^24^.

There are multiple approaches to identifying the footprints of selection. The majority of the accessible approaches use (i) the genetic difference among groups, calculated by FST (fixation index) or related statistics, (ii) the reduce in genetic variation beside footprints of selection in a population^25^. In this study, whole-genome sequencing (WGS) data from 140 goat individuals, including indigenous ecotypes from Asia (n = 75) and Africa (n = 30), as well as, commercial breeds from European (n = 35) were utilized to characterize the population structure, genetic diversity and signatures of selection analysis. Additionally, we employed nucleotide diversity (θπ) and FST statistical methods to compare Asian local goat individuals (located in West and South regions), as a population that does not undergone artificial breeding programs as yet, with European commercial goat individuals (namely Saanen, Toggenburg and Alpine breeds), to identify potential candidate genes involved in adaptation to harsh environments and milk traits. The candidate genes identified in this work may give a basis for future genome-wide association studies and research into genomic purposes of selection, especially in small ruminants.

## Results

### Aligning and SNP calling results

The average sequence coverage was 13.19 per sample covering from 5.00442X‐ to 32.46X. In addition, the total number of autosomal SNPs per individual ranged from 5,631,741 to 7,831,222 (Supplementary Table S1).

### Population structure, linkage disequilibrium decay and genetic diversity

**Figure 1:**
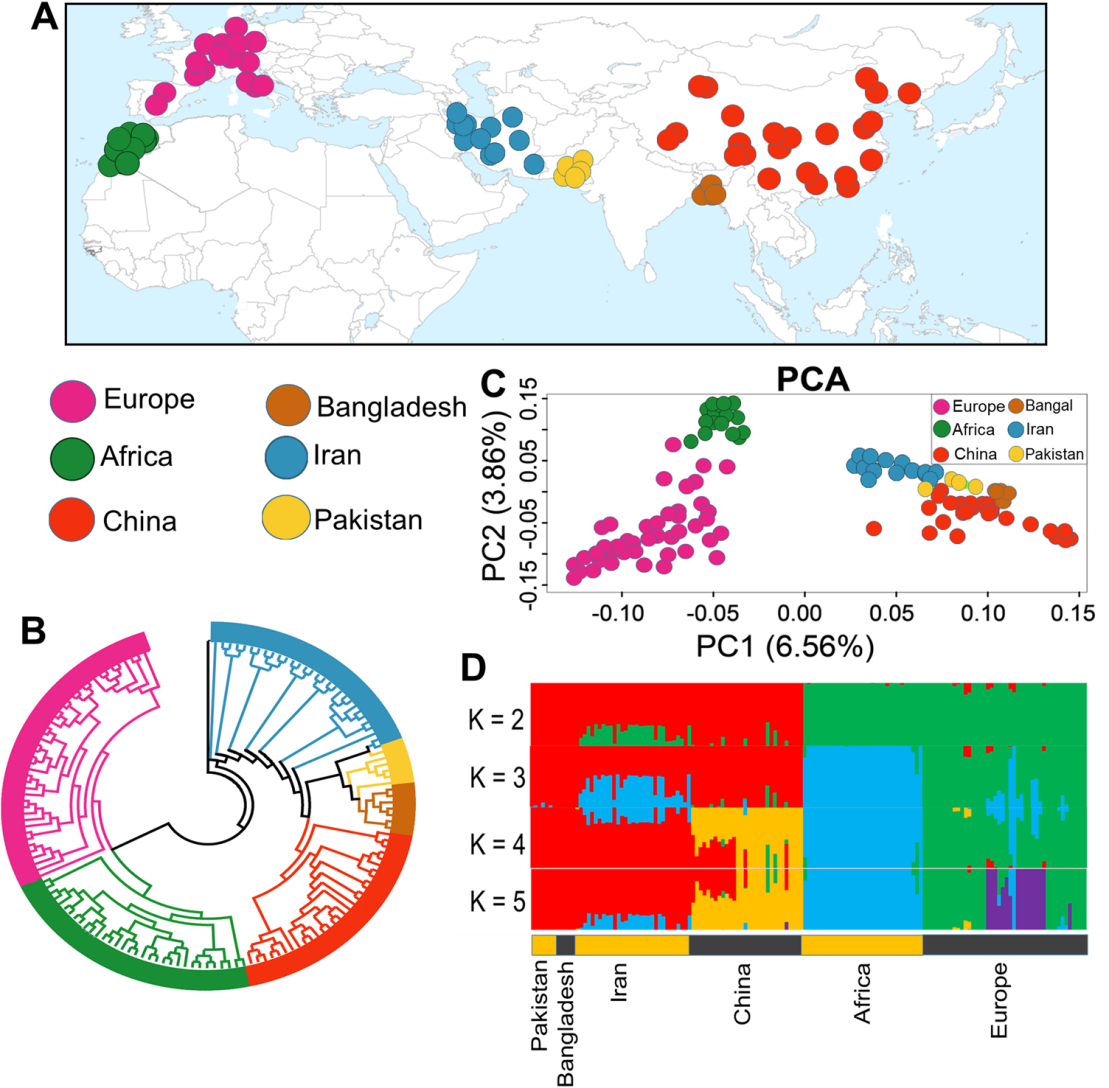
(**A**) geographical areas of studied goat groups. (**B**) a Phylogenetic tree shows evolutionary relation among different goat breeds (The figure was drawn using Samtools (version 1.31)^77^, fastree 2 tools (http://www.microbesonline.org/fasttree/) and iTOL program (https://itol.embl.de/)). C. Principal component (PC) analysis (The figure was drawn using GCTA tool (version 1.26.0)^80^ and R software environment (https://www.r-project.org/)). D. admixture analysis by assuming the ancestral numbers from K = 2 to 5 (The figure was drawn using PLINK program (version 1.9)^79^, ADMIXTURE program (version 1.3.0)^81^and R software environment (https://www.r-project.org/)).

**Figure 2:**
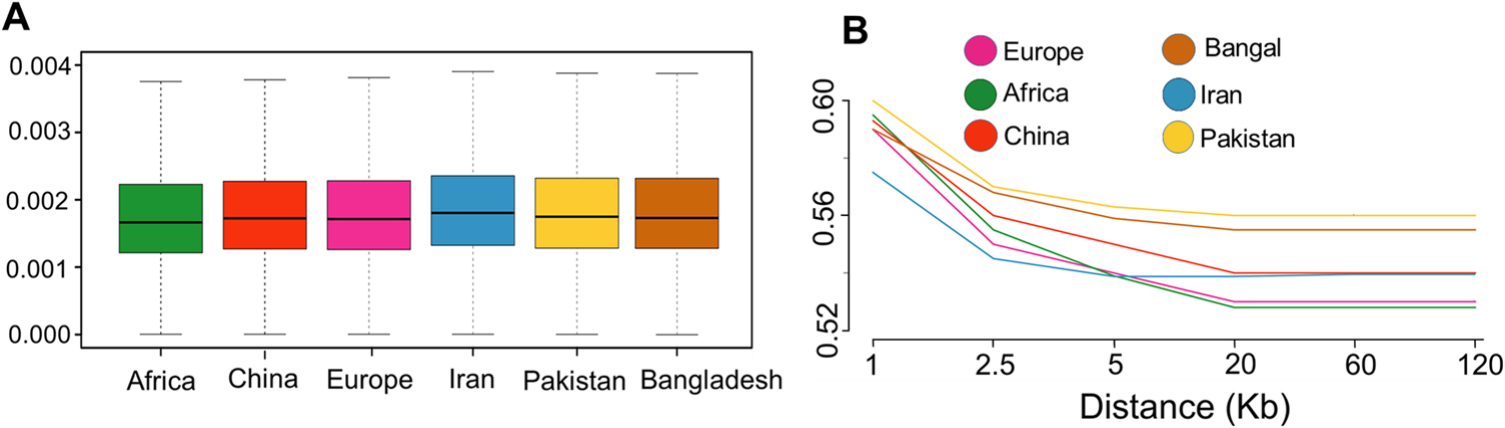
Box graphs of nucleotide diversity are calculated for different goat populations (The figure was drawn using VCFtools commands (version 0.1.17)^85^ and R software environment (https://www.r-project.org/)). The curves of Linkage disequilibrium (LD) in different goat populations (The figure was drawn using Poplddecay program (version 3.42)^82^ and R software environment (https://www.r-project.org/)).

To estimate the phylogeny relationships between studied individuals, a maximum-likelihood (ML) tree was generated. On the basis of this phylogenetic tree (Fig. 1B), the Asian native goat group was separated from other African and European populations. Focusing on the Asian group, samples from Iran, Pakistan and Bangladesh were clustered close to each other. Our findings from Admixture and principal component analysis (PCA) confirm the results of the phylogeny tree (Fig. 1C,D). The PC1 and PC2 accounted for 6.56% and 3.86% of the total genomic variance, respectively. The grouping at = *K*2 to *K*5 in admixture output showed the ancestor- ingredient for total individuals investigated (Fig. 1D). The *K* = 2 separated both African and European populations from the Asian goat population. At *K* = 3, with the lowest CV error, divides all individuals into three groups, including Asia, Africa and Europe. Ancestral proportions at *K* = 4 and *K* = 5 separated the Chinese goat individuals from the other Asian samples. In *K* = 3–5, Iranian goat samples showed some mixtures with African goat samples (Fig. 1D). In addition, we estimated the diversity (θπ) in each goat population and realized that the Iranian group has higher diversity than other goat groups (Fig. 2A). The amount of linkage disequilibrium (LD) decay between adjoining SNPs throughout the whole genome was calculated to understand the current and classical population size (Ne). The amounts of LD were presented in Fig. 2B (up to 120 kb). The r^2^ values were the highest in all considered goat groups at marker pair intervals of 1 kb [covered from ~ 0.57 (Iran) to 0.6 0 (Pakistan)] with a slow decrease along with increasing physical intervals between SNPs (up to 20 kb) and again a stable style (> 20 kb). The lowest and highest r^2^ amounts were observed in Pakistan (0.58) and Africa (0.53) goat groups at marker pairs distance of 120 kb, respectively. Furthermore, we observed that from marker pairs distance of 1 kb to ≤ 20 kb, the decrease in LD was faster in Pakistani goats than other goat groups (Fig. 2B). Concentrating on the Asian goat populations, high r^2^ values through all genomic distances were observed in Pakistani and Bangladeshi goat ecotypes that are genetically not mixed with the other Asian goat groups (Fig. 1D), whereas the lower r^2^ amounts were observed in the Iranian goat population (up to 5 kb in physical interval between SNPs), that appear genetically mixed with Africa goat group (Fig. 1D).

### Genome-wide scan for selection signatures

**Figure 3:**
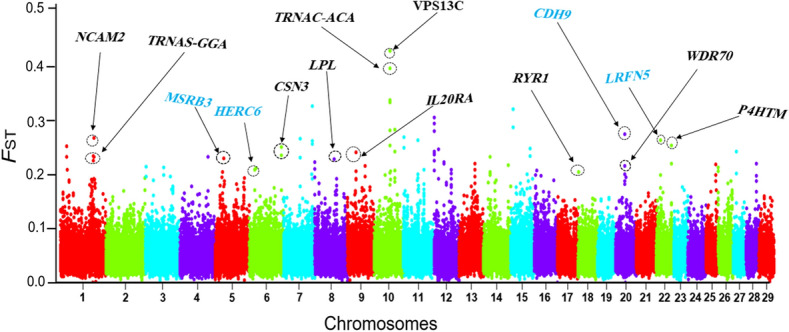
Genomic Manhattan plot of population differentiation by FST, West and South Asian indigenous goat breeds versus European goat breeds, candidate genes linked to adaptation and milk traits are shown with blue and black colors (The figure was drawn using VCFtools commands (version 0.1.17)^85^ and R software environment (https://www.r-project.org/)).

**Figure 4:**
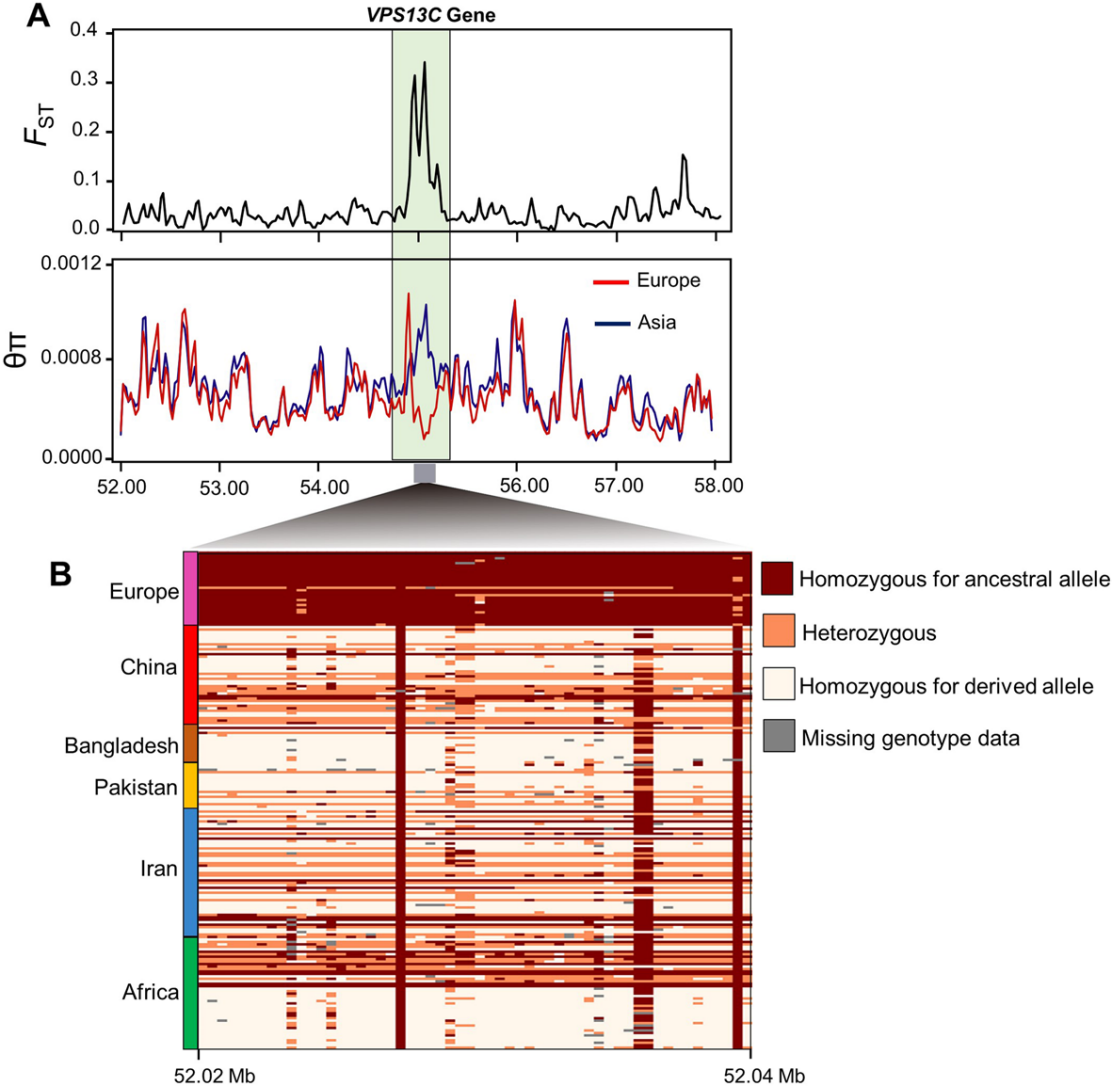
A. putative sweep area (chr. 10, 55.02–55.04 Mb) is approved by $\pi$ test (The figure was drawn using VCFtools commands (version 0.1.17)^85^ and R software environment (https://www.r-project.org/)). B. The patterns of haplotype distribution for VPS13C loci in all 140 goats. The existence of homozygosity and heterozygosity is colored in brown and intermediate brown, respectively. The absence of the derived allele is shown in white. Missing- genotyped regions or individuals are shown in gray (The figure was drawn using Beagle (version 4.0)^87^, R software environment (https://www.r-project.org/) and python scripts (our in-home script was used)).

In this study, we applied whole genome sequence data to perform comparative genome analysis between West and South Asian goat populations as one group and European goat breeds (namely Saanen, Toggenburg and Alpine) as another population to detect selection signatures that are related to various traits. We used nucleotide diversity (Pi) and *F*ST statistics to extract selection signatures remained via natural selection in local goat ecotypes or via artificial selection in commercial European goat breeds. The genomic regions that show extremely high *F*ST values (top 1% for FST) and smaller levels of nucleotide diversity (top 1% for pi) were considered to be selection signatures. Several genes that include significant *F*ST (Fig. 3 and Supplementary Table S2) and nucleotide diversity values (Supplementary Tables S3–S4) were detected in different comparisons. The top FST outlier window (55.00–55.05 Mb) includes genomic signals associated with the *VPS13C* gene on chromosome 10 (54.90–55.08 Mb), which is related with milk production traits^26^. Evidence for large negative π scores and high positive *F*ST signals at this genomic region (Fig. 4A) suggest strong positive selection at this locus. Haplotype patterns in this locus throughout all 140 goats are displayed in Fig. 4B. Haplotype pattern in this locus was completely different between commercial goat breeds (European goats) and local goat ecotypes (Asian and African goat ecotypes) (Fig. 4B).

## Discussion

To estimate genetic affinity among goat groups, we used different analysis methods (Figs. 1 and 2). The genetic divergence and PCA analysis on the basis of the genome data revealed that all Asian goat ecotypes are genetically distinct from the other studied groups. Also, the West and South Asian goat groups were distinguished as an independent population in admixture analysis (k = 3–5). Our results together indicated that the West and South Asian goat populations probably undergo distinct evolutionary processes on the basis of different geographical habituation following domestication and genetic drift, which is consistent with the previous study^27^. Furthermore, we identified several candidate genes that have been previously reported to be associated with yield phenotypes in goats and other domestic animals. The important genes attributed to adaptation to desert regions, dry weather, and milk traits are shown in Tables 1 and 2. Here we described the relationships between a number of discovered genes and adaptation and milk production traits.

**Table 1:** Identified genes by two approaches (FST and log2 θπ ratio) controlling immune responses and hot stress traits in West and South Asian indigenous ecotypes.

Statistical-method	Gene	Chromosome	Summary of gene function
FST (top 1%)-pi (top 1%)	*RYR1*	18	Immune response^48^
	*LRFN5*	21	Immune response^45,46^
FST (top 1%)	*CDH9*	20	Environmental adaptation^15^
	*MSRB3*	5	Heat stress^48^
	*HERC6*	6	Environmental adaptation^15^
pi (top 1%)	*NBEA*	12	Heat stress^15^
	*HSPB6*	18	Heat stress^29^
	*HSF4*	1	Heat stress^30^
	*MYO1A*	5	Heat stress^49^
	*RAD50*	7	Environmental adaptation^19^
	*SHQ1*	22	Environmental adaptation^15,35^
	*VPS13A*	8	Heat stress^33,34^
	*KITLG*	5	Environmental adaptation^50^
	*PGLYRP1*	18	Immune response^51^
	*IL7*	14	Immune response^47^
	*IL5*	7	Immune response^18^
	*IFNAR2*	1	Immune response^20^
	*PTAFR*	2	Immune response^28^
	*RC3H1*	16	Immune response^21^
	*STAT2*	5	Immune response^52^
	*IL23A*	5	Resistance to gastrointestinal parasite^9^
	*NOS2*	19	Resistance to haemonchus contortus in sheep and goats^53^

**Table 2:** Identified genes applying two approaches (FST and log2 θπ ratio) affective on milk trait in European dairy goats.

Statistical-method	Gene	Chromosome	Summary of gene function
FST (top 1%)-pi (top 1%)	*PDE3A*	5	Milk fat yield^62^
	*IL20RA*	9	Milk yield^63^
	*VPS13C*	10	Milk yield^26^
	*NCAM2*	1	Milk yield traits and composition traits^57^
	*TMPRSS15*	1	Milk yield traits and composition traits^57^
	*TRNAC-ACA*	10	Milk composition traits^64^
	*TRNAR-UCU*	1	Milk traits^64^
	*TRNAS-GGA*	1	Milk composition traits^64^
FST (top 1%)	*ARFGEF1*	14	Milk traits^21^
	*RBMS3*	22	Milk traits^21^
	*LPL*	8	Milk fat yield^65^
	*P4HTM*	22	Milk traits^66^
	*CCDC152*	20	Milk traits^66^
	*CSN3*	6	Milk yield traits and composition traits^60^
	*ZC3H18*	18	Milk traits^67^
	*SOSTDC1*	4	Mammary gland development^68^
	*WDR70*	20	Milk yield^69^
	*SREBF1*	19	Milk yield^70^
pi (top 1%)	*SUCNR1*	1	Milk composition traits^71,72^
	*LEPR*	3	Milk yield^16,73^
	*B4GALT1*	8	Milk traits^59,74^
	*RBM19*	17	Milk composition traits^63^
	*ABCG2*	6	Milk yield^60^

### Genes linked to desert regions, dry weather, and adaptation

Indigenous livestock animals have been genetically adapted over generations to their local environmental conditions and are desirable models to investigate the genomic processes underlying adaptability to disease and regional climates. To study this, all West and South Asian indigenous goats were combined to one group and compared to European goats. Previous studies on domestic animals have shown that the heat stress has a negative impact on the production and health, and genomic selection signatures for heat tolerance have recently become an issue for livestock species^28^. Many studies have been carried out in order to identify genomic variation associated with heat stress in goat breeds^15,16^. So, the detection of genes linked to heat tolerance can be an answer to issues connected to hot stress in the future. We identified a number of genes engaged in immune response and heat tolerance features through comparative genomic analysis between West and South Asian indigenous goats and European breeds using two high-confidence techniques (greatest 1% FST and 1% log2 θπ ratio amounts) (Table 1). We identified genes related with heat tolerance such as; *HSPB6*, *HSF4*, *VPS13A* and *NBEA* in West and South Asian indigenous goats (Table 1 and Fig. 3). *HSPB6*, an example of the most conspicuous component of the HSP group, exists on goat chromosome 18. Kumar et al.^29^ reported an association between the *HSPB6* gene and heat tolerance traits in Indian Karan Fries cattle. Heat shock protein factor 4 was annotated on goat chromosome 1. Xie et al.^30^ reported that different isoforms of this gene act as activators or inhibitors of tissue specific heat shock gene expression. The *VPS13A* (Vacuolar Protein Sorting 13 Homolog A) gene encodes chorein, considered a chief moderator of the secretion and density of blood platelets^31^. Platelet numbers alter total blood density in human beings. Heat stress raises platelet numbers and blood density, which consequently raises the danger of cerebral and coronary thrombosis^32^. This supports the assumption that the *VPS13A* gene may play a part in decreasing the danger of thrombosis through regulating platelet numbers and blood density in hot conditions. The *VPS13A* gene has been discovered in a comparative genomics study of two distinct cattle breeds from Northern and Southern China^33^. Furthermore, Ai et al.^34^ discovered that the *VPS13A* gene plays a role in southern Chinese pig adaptation to hot environments. So far, various homologues of the *VPS13* gene associated with adaptation to environmental conditions have been identified in sheep and goats. For example, *VPS13B* has been reported under selection in the Mediterranean^15^ and Chinese sheep^35^ and Moroccan^36^ and Mediterranean^15^ goats. Also, the *VPS13* C and *VPS13D* genes have been reported in tropical chicken^17^ and Mediterranean sheep^15^. Furthermore, mammalian VPS13 proteins are engaged in caring for lipids^37^. The VPS13B protein has a role in the formation and development of adipocytes^38^. In the Maasai, the VPS13D plays a role in cholesterol regulation and lactase persistence^39^. The Neurobeachin (*NBEA*) gene, which codes for the neurobeachin protein, was included in selected signatures on chromosome 12. Recently, this gene has been reported in different studies related to heat stress such as Asadollahpour Nanaei et al.^18^ in Iranian native chickens, Howard et al.^40^ in beef cattle and Serranito et al.^15^ in Mediterranean sheep and goats. Furthermore, we identified a number of genes that are likely involved in the goat immune process. Instantly, we detected a class of interleukin (*IL7*, *IL5*, *IL23A*) genes that are related to the immune process. Interleukins are expressed by leukocytes^41^. Many studies have been conducted to investigate the role of interleukins in the immune system of animals^42–44^. Another gene related to the immune system, the *LRFN5* (Leucine Rich Repeat and Fibronectin Type III Domain Containing 5) gene, is located on chromosome 21 of goats, and was found by both FST and Pi methods (top 1%). This gene is involved in immune system response, also known as B-cell mediated immunity^45^. Previous studies have reported that the *LRFN5* gene is associated with adaptation through an immune response in the South African Nguni cattle^45^ and in indigenous Iranian sheep breeds^46^. This result is according to preceding researches that reported selection signatures covering immune system genes in indigenous sheep and goats^9,47^.

### Candidate genes associated with milk production traits

The quantity of milk, proteins, and milk fat are particularly important traits in dairy livestock. However, little is known about the area of the genome that controls these important traits in goats. We detected positive selection signatures for milk traits through comparing the genomes of Asian (West and South) indigenous goats with those from European. Whole-genome re-sequencing data from indicative European breeds (namely Saanen, Toggenburg and Alpine) and local native breeds from West and South Asian goats provided a complete list of genomic diversity. Due that European breeds are considered as among the greatest milk yielding in the world^54^, comparative genome analysis of Asian local breeds and European goats is a desirable approach to detect genomic diversity in milk yield phenotypes. To achieve this purpose, we applied two statistical techniques, including comparisons between two groups and within a group. Protein-encoding genes identified by both FST and nucleotide diversity were reported in Supplementary Tables S2 and S4. We further found a number of genes linked to milk production traits in goats and other ruminants (Table 2). The most notable of them is the *VPS13C* (Vacuolar Protein Sorting 13 Homolog C) gene that covered the top FST outlier window (on chromosome 10; 55.02–55.04 Mb). Also, low π scores proposed potent positive selection at this location in European goats (Fig. 4A). A number of previous studies have reported the association of the *VPS13C* gene with milk production traits in goats^26^ and cattle^55^. A previous study stated that the *VPS13C* gene motivates glucose homeostasis for high milk production in cattle^55^. Furthermore, the different haplotype patterns of the *VPS13C* gene (chromosome 10; 55,075–55,125 kb) in local goat populations, including Asia and Africa vs European goat population (namely Saanen, Toggenburg and Alpine) suggest the *VPS13C* gene as a candidate gene related to milk trait in commercial dairy goats. Neural Cell Adhesion Molecule 2 (*NCAM2*) and Transmembrane Serine Protease 15 (*TMPRSS15*) genes were identified as candidate genes in a selective sweep region belonging to goat chromosome 1 (top 1% cutoff of FST and Pi methods). Previous works have reported the associations of the *NCAM2* and *TMPRSS15* genes with fat, protein, and milk yield^56,57^. Another milk-related candidate gene, Kappa-casein *CSN3*, was identified in a region located on goat chromosome 1 (top 1% cutoff of FST). The *CSN3* gene is an important candidate gene that impacts milk yield traits. Catota-Gómez et al.^58^ have reported genomic mutations of *CSN3* are extremely related to the milk protein percentage. Many independent researchers have reported that its polymorphisms are related to milk yield features (fat, protein and milk production)^59^ and compound traits (protein and fat percentages)^59,60^ in different dairy cattle breeds. The ATP Binding Cassette Subfamily G Member 2 (*ABCG2*) gene was detected as a candidate gene in a selective sweep region belonging to goat chromosome 6 (top 1% cutoff of Pi). A previous study has reported that the *ABCG2* gene is strongly related to milk production and composition traits^61^.

## Conclusions

In this study, we discovered several novel and also previously known candidate genes related with milk production traits and adaptability to dry and heat tolerance in goats that can be important for breeding designs. Nevertheless, more research is required to confirm phenotype-genotype connections of the detected genes in this work.

## Methods

### Genome sequences, short read mapping and SNP calling

Whole genome sequence data of 140 goats from Iran (n = 36), China (n = 30), Pakistan (n = 4), Bangladesh (n = 5), Africa (n = 30) and Europe (n = 35) were downloaded from public sequence databases (https://trace.ncbi.nlm.nih.gov/Traces/sra) (Fig. 1A and Supplementary Table S1).

After quality processing of the raw data, the Burrows–Wheeler Aligner (BWA) program (https://sourceforge.net/projects/bio-bwa)^75^ was utilized to map the sequence data toward the reference assembly of goat genome (ARS1, GCF_001704415.1)^76^.

We applied SAMtools commands^77^ to transform sequence alignment map (SAM) files to binary alignment map (BAM) files. Applying Picard commands (https://github.com/broadinstitute/picard), PCR duplicates were removed from the bam files. Later, in order to raise the quality score for each base, recalibration of the base quality scores was carried out using BaseRecalibrator and IndelRealigner commands from Genome Analysis Toolkit 3.4 (GATK)^78^. Lastly, SNP discovery and SNP Filtration were done using the UnifiedGenotyper and the Variant Filtration commands the GATK program.

### Population structure and genetic ancestry analyses

We used the ML technique to create an evolutionary tree. We applied vcf2fq in vcfutils.pl from Samtools to convert filtered VCF files into FASTA files. The converting of filtered VCF files into FASTA files was done by applying vcf2fq into vcfutils.pl in Samtools and the following utilized FastTree 2 tools to construct a phylogenetic tree. We used the iTOL program (https://itol.embl.de/) for drawing the evolutionary picture. Before the genetic structure analysis, the SNPs data were pruned for LD in PLINK^79^. Admixture and the PCA were done on pruned SNPs for LD. Genome wide complex trait analysis (GCTA)^80^ on the basis of SNP genotypes was utilized to specify genetic variation between all goat populations. To study the realizable genomics admixture among groups, we utilized the admixture model applied in the ADMIXTURE program^81^, applying several values of K (from 2 to 5) and 10,000 iterations. The decay of LD was computed using Poplddecay program^82^ for different genetic distances between SNP pairs (1, 2.5, 5, 20, 60 and 120 kb).

### Statistics to detect selection signatures

Two complementary approaches were applied to explore selection signatures. We computed the genome-wide weighted FST^83^, because this method is a suitable scale of mean genetic variation between populations having different sizes^84^. In addition, we calculated diversity (θπ) employing VCFtools commands (‐ window‐pi 50,000 ‐‐window‐pi‐step 25,000)^85^. Sliding window analyses at the level of the genome were done with a step size of 25 kb and a window size of 50. The log2 (θπ South and West Asia/θπ Europe and θπ Europe/θπ South and West Asia) and mean FST values of SNPs per window were computed. Goat gene IDs that covered all candidate regions were extracted from Ensemble annotation^86^. To display the specific genotypes patterns of the putative selective region (VPS13C gene on chromosome 10, 55,02–55, 04 Mb), we used Beagle to phase the SNP genotypes and construct haplotype patterns between different populations^87^. Finally, we showed specific genotypes patterns in a heatmap using python scripts and R software environment.

## Supplementary Information

Supplementary Table S1.

Supplementary Table S2.

Supplementary Table S3.

Supplementary Table S4.

## Data Availability

The compiled VCF file is available from the corresponding author upon reasonable request. The other data produced in this work have been presented as supplementary information with this manuscript. The raw sequence data used in this study were downloaded from the public sequence database (https://trace.ncbi.nlm.nih.gov/Traces/sra).
